# Frontline realities in South African disaster response: Social workers’ perspectives on practice challenges

**DOI:** 10.4102/jamba.v18i1.2055

**Published:** 2026-09-03

**Authors:** Lehlogonolo K. Poopedi, Nontembeko J. Bila

**Affiliations:** 1 Department of Social Work and Criminology, Faculty of Humanities, University of Pretoria, Pretoria, South Africa

**Keywords:** disaster response, frontline social workers, disaster social work, South Africa, practice challenges.

## Abstract

Social workers increasingly integrate disaster work into their practice, recognising it as a developing field within social work. For years, social workers have provided promotive, preventative and treatment services during disasters to ensure community well-being. The increasing demand for social workers in disaster management is driven by a world that is becoming more disaster-prone, requiring effective responses to mitigate their impacts. However, various challenges hinder the effective delivery of disaster social work services. Based on the latter, this article explores the multifaceted challenges faced by disaster frontline social workers and the implications for the practitioners and service delivery. This article reports on findings from the qualitative component of a broader mixed-methods study. Data were collected through semi-structured interviews using an interview schedule, informed by the perspectives of 21 disaster frontline social workers from South Africa, specifically the provinces of Gauteng and KwaZulu-Natal.

**Contribution:**

The findings reveal that both natural and human-made disasters present unique challenges that require social workers to be adaptable and resilient. Key challenges include inadequate resources, overwhelming caseloads, safety concerns, insufficient training, role confusion and lack of cooperation from both community members and stakeholders. Addressing these challenges through targeted training, clearer definitions of roles in disaster response and strengthened intersectoral collaboration is essential for fostering a resilient and responsive social work force during disasters.

## Introduction

Disasters continue to increase in both frequency and intensity, posing a significant threat to the lives and livelihoods of people globally (Bauwens & Naturale 2017; Boetto 2024; Pourazar 2017). Social workers are among the social service professionals directly engaged in mitigating the consequences of disasters to develop community strengths and promote long-term recovery post-disaster (Dominelli 2015; Machimbidza, Nyahunda & Makhubele 2022; Shokane 2019; Sweifach, Linzer & LaPorte 2013). In this regard, social workers play a pivotal role in supporting vulnerable populations, drawing from a long history of engagement in disaster-related interventions (Drolet et al. 2024; Hay & Pascoe 2021; Nikku 2015).

The profession of social work is grounded in theory and practice, guided by the global agenda for social work, which recognises how inequalities and unsustainable environments affect people (Hay & Pascoe 2021; Matlakala, Makhubele & Nyahunda 2022). Disasters adversely affect individuals’ health, livelihoods and socio-economic standing, which are issues that social workers are mandated to address within their overall scope of practice and endeavours to enhance human well-being (Nyahunda & Ncube 2025; Shokane 2019; Youssif-Hay, Tudor & Maidment 2024). Based on the latter, social workers have a crucial role to play in ensuring the welfare of individuals, families, groups and communities, even in the midst of disasters (Cuadra 2015; Drolet et al. 2024; Machimbidza et al. 2022; Shokane 2019). Disaster social work is the practice of social work during disasters, focusing on supporting and strengthening individuals’ and communities’ ability to cope with the aftermath (Mohinuddin 2020). Social work practice is placed at the nexus between government role players and communities, promoting and facilitating resilience (Cuadra 2015; Matlakala et al. 2022; Nyahunda & Ncube 2025).

Social workers are uniquely positioned to respond to disasters because of their human rights-based approach and experience of working with vulnerable individuals and communities, including those affected by both natural and man-made disasters (Hay & Pascoe 2021; Matlakala, Nyahunda & Makhubele 2021; Okafor 2021). The nature and design of social work further render it particularly suitable for disaster response and relief because of its ecological approach, which considers individuals within their broader environmental context, and its holistic perspective, which facilitates the identification of strengths, resources and challenges experienced by disaster victims and survivors (Alston, Hazeleger & Hargreaves 2019; Dominelli 2015; Drolet et al. 2024; Sim, He & Dominelli 2021). These aspects are not always the primary focus of other professionals involved in disaster response and particularly distinguish social work from other professional disciplines involved in disaster response (Alston et al. 2019). Social workers also contribute uniquely by promoting continuous person-centred and community-based care, advocating for the inclusion and meaningful participation of those affected in all aspects of decision-making and engaging with vulnerable and hard-to-reach communities to help build their adaptive capacity (Alston et al. 2019; Hay & Pascoe 2021; Huang, Fu & Wong 2014; Machimbidza et al. 2022). For instance, social workers are trained in assessment and intervention methods and skills that can be applied cross-culturally and across diverse disaster contexts (Hay & Pascoe 2021). Additionally, the uniqueness of their practice lies in their ability to build rapport and trusting relationships between communities and organisations, which is essential to foster cooperation in the aftermath of a disaster (Hay & Pascoe 2021). Their contribution is also critical for mitigating the consequences of disasters and promoting sustainable recovery by building on lessons learned from routine and novel interventions (Cheung 2015; Federal Emergency Management Agency [FEMA] 2011). Moreover, their involvement in disaster preparedness, response and recovery has been widely acknowledged as essential to effective disaster management systems (Cuadra 2015). However, working in the frontline setting has continuously been associated with various challenges, which hinder effective service delivery and negatively affect disaster frontline social workers (Brooks et al. 2016; Kranke et al. 2020; Seng et al. 2021; Sweifach et al. 2013)

### Problem statement and rationale

Despite the widely recognised contributions of social workers in responding to the impacts of various disasters in society (Alston et al. 2019; Drolet et al. 2024; Kranke et al. 2022; Matlakala et al. 2021), research documenting the challenges of service provision by disaster frontline social workers in South Africa remains limited and mainly dominated by global perspectives (Nyahunda & Ncube 2025; Shokane 2019). The available literature on social work practice during disasters, including the specific challenges practitioners encounter, remains scant in the South African context. Similarly, the majority of social work studies on the impact of disasters on practitioners and on their interventions have mainly focused on affected populations while neglecting the systemic barriers that constrain effective social work service delivery during disasters.

Based on the latter, understanding the challenges encountered by social workers at the front lines of disaster response efforts is crucial for informing how social work service delivery can be improved to ensure the effective provision of support to those affected by disasters and to strengthen the overall resilience of the disaster response system. Additionally, this is important because South Africa has become disaster prone, thus suggesting a growing and potentially sustained demand for social work services during disasters (Pourazar 2017). Furthermore, Youssif-Hay et al. (2024) emphasise the importance of social workers being aware of and responsive to the impacts of disasters. Youssif-Hay et al. (2024) further highlight that research on social work practices in both natural and man-made disasters is essential to improving these efforts. Therefore, exploring and documenting the challenges frontline South African social workers face in disaster response, and their implications for practitioners and service delivery are equally valuable as it can also heighten awareness of the challenges inherent to disaster social work service delivery in South Africa while also providing recommendations to address these challenges in order to strengthen practice.

### Challenges experienced by social workers in disaster response

Significant challenges face disaster frontline responders, including social work practitioners, making it difficult for them to execute their roles and responsibilities during and after disasters (Matlakala et al. 2021).

Social workers often need to navigate personal challenges stemming from their experiences on the front lines of disaster response, as disasters pose acute shared trauma for both practitioners and those they serve (Banks et al. 2020; Huang et al. 2014). In this regard, social workers get exposed to the same mental stressors and societal shifts as the public while catering to the needs of service users and navigating their own fear of exposure to harm posed by the disaster (Dominelli 2015; Huang et al. 2014). Social workers are particularly susceptible to stress and burnout, which negatively affect their physical and psychological well-being and job productivity (Rai & Rammanohar 2023). The likelihood of practitioners experiencing high stress and burnout can also be attributed to the inherently stressful nature of social work as a helping profession, suggesting that practitioners are likely to experience these challenges (Seng et al. 2021). This is also evidenced by several studies (Alston et al. 2019; Kranke et al. 2022; Waterman et al. 2018) that show that a significant proportion of frontline workers experience physical and psychological challenges, including diagnosable mental health conditions, because of disaster exposure. Providing services to those affected by disasters further places an emotional toll on frontliners, leading to feelings of insecurity and hopelessness that often disrupt their full functioning (Kranke et al. 2022; Sweifach et al. 2013).

Engaging in disaster work further has social and relational implications for social workers, as those with families experience intensified work–family balance conflicts (Huang et al. 2014). In a similar vein, Dominelli (2021) recognises how social workers often engage in disaster response at the expense of their own family life, with some even dying while performing their work. For example, during the COVID-19 pandemic, most practitioners were isolated and, in some cases, cut off from their main sources of social support, including family gatherings and time spent with friends (Bennet 2021). Experiencing stigma for inadvertently compromising their loved ones and being pressured to resign by family members who are fearful of the perceived risks and dangers of working in disaster contexts is also common for practitioners (Cheung 2015).

The threatened loss of these social resources and the fear of one’s own immediate family’s demise provoke psychological distress, reducing the capacity of practitioners to cope (Heanoy & Brown 2024).

In the workplace, the experiences of frontline social workers in most practice contexts share common challenges, including addressing organisational disruption and disarray, and tackling poor or inadequate professional preparedness and resource shortages (Banks et al. 2020; Boetto 2024; Matlakala et al. 2021; Van Heugten 2013). Numerous work-related factors, including prolonged shifts, lack of physical or psychological safety, perceived job security, work-related bullying and lack of support, mainly contribute to elevated stress among frontline practitioners (Matlakala et al. 2021). Owing to these, social workers are often required to adapt to various disaster contexts to ensure the effectiveness and continuity of services to disaster victims and survivors, without adequate resources, a clear scope of practice and relevant disaster training (Banks et al. 2020; Cuadra 2015; Dominelli 2021; Matlakala et al. 2021; Shokane 2019). Working in environments with limited financial and material resources further makes it difficult for social workers to meet both the immediate and long-term needs and expectations of those affected by disasters (Rai & Rammanohar 2023). Insufficient professional training in the workplace, compounded by the lack of a clear scope of practice, also results in a lack of self-confidence and limited competence among practitioners, hindering their ability to perform their roles and tasks effectively during disasters (Sim et al. 2021).

Social workers, on the other hand, face ethical dilemmas arising from conflicts between professional social work values, client needs, organisational demands and limited resources (Dominelli 2015; Matlakala et al. 2021; Van Heugten 2013). Social workers are also expected to provide the best possible support to disaster-stricken individuals and communities while simultaneously managing pre-disaster caseloads, which Sweifach et al. (2013) note contribute to the displacement of personal concerns and a strong inclination to focus on others before taking care of oneself. Collectively, these challenges increase stress levels and place social workers at high risk of burnout, which is often linked to deterioration in their mental and physical well-being (Albott et al. 2020; Drolet et al. 2024; Kranke et al. 2022). Burnout can also manifest as psychological and physical exhaustion, which can deplete social workers’ ability to provide services effectively and remain empathetic to clients, leading to withdrawal or leaving their employment (Cheung 2015; Soto 2024; Van Heugten 2013). Hearing the stories of victims and survivors and witnessing trauma in the form of injuries, death of clients and other frontline workers can contribute to symptoms of compassion fatigue, feelings of sadness and vicarious trauma (Boetto 2024; Brooks et al. 2016; Kranke et al. 2022; Sweifach et al. 2013).

In the broader community, there is often pressure on social workers to take action when disasters occur in their communities, which leads to an overextension of themselves as they try to assist too many people, which eventually leads to burnout months into the post-disaster period (Alston et al. 2019; Dominelli 2015; Matlakala et al. 2021). The physical impacts of disasters on the environment can at times disrupt the environments in which practitioners work, leading to either cramped or open-plan workplaces that lack privacy and compromise client confidentiality, which is of utmost importance in social work (Van Heugten 2013). Moreover, the environmental damage to roads and infrastructure makes it difficult, if not impossible, for social workers to conduct home visits (Van Heugten 2013).

Overall, the literature demonstrates that frontline service providers, including social workers, bear a significant burden in responding to the challenges and consequences of disasters. Overall, these challenges have a ripple effect, compromising effective service provision and having long-term implications for workplace morale, patient safety and quality of care (Albott et al. 2020; Kranke et al. 2022).

## Research methods and design

### Goal and objectives

Goal: This study aims to explore and describe the multifaceted challenges experienced by disaster frontline social workers when providing services to those affected by disasters and to examine the impact of these challenges on their practice and service delivery.

The study has three objectives:

- To explore and document the challenges encountered by frontline social workers when providing services during disasters.
- To explore and describe the impact of these challenges on the practice of and provision of social work services during disasters.
- To provide recommendations for addressing these challenges in order to enhance the effectiveness and quality of social work service provision during disasters.

### Theoretical framework

The study was underpinned by the ecological-systems theory, which is a holistic theory of human development that focuses on the interdependent relationships between individuals and the environments in which they exist (Mbedzi 2019; Neal & Neal 2013). The theory comprises five interrelated environmental systems: The micro, meso, macro and chronosystems. These represent either proximal settings where individuals directly interact or broader, distal systems that indirectly influence development and functioning (Ettekal & Mahoney 2017). These systems collectively account for the complex, layered contexts that shape individual experiences over time. The ecological-systems theory was found suitable for this study as it shifts from an individualistic perspective to a multisystemic understanding of the person-in-environment (Mbedzi 2019). The theory further recognises development and functioning as shaped through reciprocal interactions between individuals and various factors across the previously mentioned systems, which may present either in enabling or in constraining ways (Crawford 2020; Ettekal & Mahoney 2017). By adopting this theoretical lens, the researchers could view disaster frontline social workers not in isolation, but as embedded within interconnected systems, including but not limited to families, workplaces, the communities they serve and the broader environmental and organisational contexts within which they operate. This theory also served as a useful lens through which the researchers could explore and better understand how challenges emanating from these systems, as well as their interactions, directly or indirectly, influence social workers and their service provision to those affected by disasters.

### Research approach and paradigm

The study was underpinned by the pragmatist paradigm, which involves collecting and analysing both quantitative and qualitative data, emphasising the practical application of knowledge and prioritising the research question over adherence to a single methodological tradition (Delport & Fouchè 2011). Pragmatism is primarily based on the premise of solving practical problems in the real world and recognises that no single approach can fully capture complex local realities; thus, it supports the use of multiple methods to generate findings that are both contextually relevant and action oriented (Maarouf 2019).

The study employed a mixed-methods research approach, following a sequential exploratory design, that begins with an exploratory qualitative phase and then sequentially moves to a quantitative phase (Roomaney & Coetzee 2018). The mixed-methods approach refers to a research process in which qualitative and quantitative data collection and analysis methods are integrated to best meet the research objectives and address the research problem fully within a single study (Harrison, Reilly and Creswell 2020; Plano-Clark & Invankova 2015). However, this paper focuses on the findings and analysis of the qualitative data collected during the first phase of the study. The qualitative research was exploratory, enabling the researchers to gain initial insights into social workers’ experiences of the challenges encountered in disaster contexts, particularly their impact on practitioners and on service delivery to disaster-stricken individuals, groups and communities. Exploratory qualitative methods were used to identify codes and qualitative themes (Delport & Fouchè 2011).

### Research design

The study employed a collective case study design, also referred to as a multi-site case study, which involves more than one case that may or may not be physically collocated but share a common link (Cresswell & Plano Clark 2017). The central tenet of this design mainly lies in the need to explore an event or phenomenon in-depth (Cresswell & Plano Clark 2017). Employing this design facilitated an in-depth understanding of the challenges disaster frontline social workers encounter in providing services to those affected by disasters in their natural, real-life contexts (Halkias & Neubert 2020). This design further enabled an in-depth, multifaceted exploration of a phenomenon and yielded rich insights by providing evidence from multiple cases (Yin 2014).

### Study population and size

The study primarily targeted all social workers employed by selected metropolitan municipalities in the Gauteng and KwaZulu-Natal provinces, with a high concentration of social workers actively engaged in disaster management. Furthermore, the two provinces were selected because they had demonstrated vulnerability to various types of disasters and had been documented as having high disaster risk profiles (Edoun & Bakam 2022; Khanyile & Fatti 2018; Mkhulisa 2017; National Disaster Management Centre [NDMC] 2024). However, only 21 frontline social workers, employed by the City of Johannesburg and the eThekwini North and South metropolitan municipalities, participated in the study because data saturation was reached.

### Sampling

Non-probability sampling, more specifically purposive sampling, was employed. Purposive sampling refers to the process of selecting a sample with a specific purpose in mind (Palinkas et al. 2015).

The participants had to meet the following criteria to be included in the study:

- Be willing and available to participate in the study. There were no limitations with regard to age or gender.
- Have at least 1 year of experience in rendering services within the participating municipality.
- Have served as a frontline service provider during disasters.
- Be registered with the South African Council for Social Service Professionals (SACSSP) with a recognised bachelor’s degree from a South African university.
- Be able to speak and understand English.

### Data collection

In-depth, semi-structured interviews were conducted to collect the qualitative data. These consisted of a dialogue between the researchers and each participant, guided by a flexible interview schedule and supplemented by follow-up questions, probes and comments (DeJonckheere & Vaughn 2019). This enabled the researchers to gather rich, in-depth and contextually relevant data. Interviews further allowed for flexibility by enabling the researchers to adequately explore the thoughts, feelings and beliefs about the challenges encountered in providing social services to support disaster victims and survivors (DeJonckheere & Vaughn 2019; Neuman 2012).

### Data analysis

Qualitative data from the semi-structured interviews were analysed in accordance with the six steps of thematic analysis as described by Terry et al. (2017), namely familiarisation, coding, searching for themes, reviewing themes, identifying and naming themes and writing the report.

### Ethical considerations

Ethical considerations, including the avoidance of harm, informed consent, voluntary participation, no deception of respondents, no compensation of respondents and adherence to anonymity and confidentiality, as well as debriefing of participants, were adhered to (Babbie 2017; Strydom 2011). The study obtained full ethical clearance from the University of Pretoria Faculty of Humanities research ethics committee with protocol no: HUM010/1122.

## Results

### Challenges experienced by social workers in disaster response

This section outlines the study’s findings on the key challenges frontline social workers encounter in responding to disasters.

## Discussion

### Lack of resources

The participants identified the lack of resources, particularly transport, operating budgets and supplies for victims and survivors, as the primary challenge encountered during disaster response. They reported often navigating disaster response with limited resources, making it difficult for them to adequately address the immediate and long-term needs of disaster victims. A social worker explained how resource constraints affect service delivery, saying:

‘You know, sometimes it’s so hurtful. Let’s say, for example, there’s a disaster, there are maybe floods, and we are waiting for the cars or something like that. And you find that maybe in that time, we didn’t have cars to reach that place. So I think, like the resources, it really affects me. And also the people who are affected by the floods.’ (Participant 18, female, social worker)

Another described the challenges, saying:

‘… servicing the community with limited resources and then trying to manage the needs of the people who are displaced. Trying to manage their behaviour and also trying to give them that hope that everything is going to be okay, even though sometimes you see that there are no resources.’ (Participant 10, female, social worker)

The lack of resources was reported to severely limit the ability of social workers to adequately cater to the rising needs of victims and to fulfil their roles both as desired and mandated. Participants reported feeling demoralised by this and by being forced to use their personal resources (such as their own vehicles) to fulfil expectations and somewhat compensate for their department’s shortcomings. Several authors (Madlah et al. 2023; Matlakala et al. 2021; Rai & Rammanohar 2023) similarly note that limited resources undermine effective service delivery and the overall success of social work interventions for those affected by disasters. In this regard, social workers often have to address resource shortages by using their own resources to render services (International Federation of Social Workers [IFSW] 2020). In a study investigating how different aspects of collaboration influence individual performance among individuals and organisations involved in major wildfire disasters in Sweden, Guerrero et al. (2023) identified resources among key capacity aspects that are critical for equipping individuals to perform in collaborative systems and, in particular, disaster situations. From an ecological-systems perspective, resource constraints emanating from the exo-system adversely impact and directly shape service delivery in the micro-system. These also contribute to feelings of frustration and demoralisation, which can compromise both their well-being and the comprehensiveness and effectiveness of service delivery to clients in the micro-system (Crawford 2020; Neal & Neal 2013).

### Heavy caseloads

The participants reported that disaster events added to their existing caseloads as they were required to take on additional disaster-related responsibilities, alongside organisational pressure to meet performance targets.

This creates significant pressure and frustration, requiring them to make multiple compromises, including working overtime at the expense of their family time. A social worker described the frustration of carrying this extra work:

‘We have to work overtime. It’s a challenge because time that’s dedicated to my family also … then it means that I cannot take it for myself and my family. The people that we serve at the hall are not there during the week. If we were to reach them, we would have to go and visit the halls on the weekends, Saturdays and Sundays. I’m not going to be able to cope with the workload. Remember, during those disaster times, we still have our normal caseload, so we have to juggle that work with our normal work, it can be a lot especially if we are not trained, because it means that we have to now start from scratch, to research now what we do, before we receive from the top.’ (Participant 8, female, social worker)

Another reiterated the heavy workload expected of them, saying:

‘For me, that taxed me the most because it’s not to say, okay, fine, deal only with your workload. No, no, deal only with your flood. You had to deal with all your other caseloads. That for me was not okay with regards to mental because whether we liked it or not, we’re answerable, we’re going to be disciplined if we didn’t make our targets. So, we were exhausted at that time. I think we were so frustrated.’ (Participant 1, male, social worker)

Disaster events create additional demands and tasks for social workers, significantly increasing their workloads (Matlakala et al. 2021). A study by Cederbaum et al. (2022) similarly highlighted that social workers are often assigned additional roles during disaster response and pressured to implement additional disaster management protocols and standards. These increased caseloads heighten the likelihood of fatigue and burnout (Huang et al. 2014; Kranke et al. 2020). Additionally, they disrupt practitioners’ daily schedules, making it difficult for them to maintain self-care activities and increasing their susceptibility to stress (Kranke et al. 2020). The findings reflect exo-system impacts on the micro-system, with workplace deficiencies beyond the practitioners’ control attributed to the high caseloads in the micro-system. As noted in the findings, these caseloads and organisational pressures, which compounded over time, also have long-term implications for practitioners’ well-being, compromising their micro-system functioning, including their relationships with significant others (Neal & Neal 2013; eds. Newman & Newman 2020).

### Safety of social workers

The study found that the safety of social workers was significantly compromised during disaster response, particularly in high-risk communities. In this regard, social workers often had to navigate such environments without adequate support from other key disaster management counterparts, such as the South African Police Service (SAPS). An interviewee described a dangerous situation they encountered while in the field:

‘I remember when we were going to these shacks where people got flooded. I remember one day, one man screamed, and he said, We must just block the way so these social workers mustn’t go out. Because they are not providing us with what we need.’ (Participant 19, female, social worker)

Another explained that the role of the social worker was often misunderstood, leading to conflictual situations when working among disaster-impacted communities:

‘Sometimes they even threaten us by saying, “Why are you here?” What are you bringing? All those things? So, what about accommodation? Where are you going to take us to so there are a lot of issues, and for us, the purpose is that we are not there to provide as yet as part of the accommodation. Sometimes we are there so that you can get the essence of what happened, and then you inform relevant stakeholders.’ (Participant 1, male, social worker)

Several authors (Brooks et al. 2016; Madlah et al. 2023; Quevillon et al. 2016) have similarly recognised that disasters pose direct threats to the safety of responders. This is often the case in volatile environments where the threat of harm to social workers increases, requiring strict adherence to safety and security protocols (Madlah et al. 2023). The perceived risk of disasters to oneself and worries about personal safety and inadequate safety measures also cause anxiety and are linked to post-traumatic stress disorder (PTSD) among frontline practitioners (Brooks et al. 2016). A study conducted by Rai and Rammanohar (2023), to analyse the challenges experienced by social workers during disaster response and recovery, in contrast to this study, found that safety concerns were less commonly reported in comparison to challenges such as resource constraints, coordination issues, training deficiencies and emotional stress. Despite this, Rai and Rammanohar (2023) highlighted that safety concerns remained significant among affected frontliners and had a substantial impact on their mental well-being.

Personal safety concerns in the micro-system reflect the influence of broader socio-economic community vulnerabilities that have accumulated over time (chrono-system) and are exacerbated by disasters, creating high-risk response environments for participants (Brakenhoff & Slesnick 2015; eds. Newman & Newman 2020). Meso-system coordination gaps, including inadequate organisational safety protocols, further contributed to these concerns (Neal & Neal 2013).

The participants further alluded to the lack of safety measures, such as the provision of personal protective equipment (PPE) to social workers, when responding to disasters:

‘We don’t have any PPE to use during floods. You don’t have boots. You don’t have anything. You use your own thing. You wear your clothes. And that is very stressful … And you’ll see other departments coming well equipped with their PPEs, and with you is just you and your name badge.’ (Participant 5, female, social worker)

‘I know it’s been a big hassle in terms of debating around PPE, and it is not being provided. So, the disaster team would come and they’re fully clothed, but a social worker just comes there in their normal clothes. Normal shoes walking over the rubble and stuff like that.’ (Participant 3, female, social worker)

Cederbaum et al. (2022) associate the failure of workplace policies to explicitly acknowledge social workers as frontliners as one of the key reasons for their exclusion from PPE. In a study exploring the experiences of social and auxiliary workers during the pandemic, Gunhidzirai, Chamisa and Ruzungunde (2022) similarly found that practitioners experienced critical shortages of PPE. Gunhidzirai et al. (2022) noted that this challenge had significantly impacted practitioners’ capacity to provide services effectively and undermined their well-being. A few other studies (Cabarkapa et al. 2020; Gunhidzirai et al. 2022; Kranke et al. 2022) also recognised that this challenge further heightened the fear of contracting and spreading the virus to their families and increased work stress among practitioners. Surprisingly, Brooks et al. (2016) assert that in instances where PPE is provided, concerns about its quality arise, which also exacerbate stress levels and anxiety among practitioners (Brooks et al. 2016). Nonetheless, PPE is crucial in enabling practitioners to reach more victims during disasters despite its limitations, such as making meaningful eye contact through foggy safety goggles (Crawford 2020).

The participants further reported making personal means to substitute non-provision and the availability of PPE:

‘I even resorted to having extra flat shoes in the car so that I can protect myself. Because the city is not prioritising our safety. To answer your question, I don’t think we have any available protective measures.’ (Participant 3, female, social worker)

The participants in the study by Gunhidzirai et al. (2022) similarly reported using whatever materials were available to substitute for unavailable PPE and to continue services. However, unlike the current study, the participants in Gunhidzirai et al. (2022) received PPE donations through external initiatives led by well wishers and companies engaged in social responsibility. The lack of PPE reflects how, over time (chrono-system), persistent meso- and macro-system gaps in the provision of required PPE for practitioners have led practitioners to rely on their personal resources (Neal & Neal 2013; eds. Newman & Newman 2020). Practitioners do this not only to safeguard themselves against disaster risks but also to maintain service delivery to those affected by disasters in the micro-system, even though this is an employer obligation explicitly mandated by the IFSW (2020).

### Lack of internal and external disaster management training

The lack of disaster management training was also identified as a significant concern, as participants reported being inadequately prepared and ill-equipped with the knowledge and skills necessary for effective disaster response. This contributed to their uncertainty about how to best respond when confronted with a disaster, reducing their self-confidence when responding, as the following direct quotes from several social workers illustrate:

‘The problem is that no one prepares you for all those things. It’s like you are thrown into a deep sea, having to swim on your own. You have to use your discretion as well as how to handle things … we end up not knowing what to do when there are disasters.’ (Participant 19, female, social worker)

‘No, nothing, I don’t even want to talk much about it. Here you swim or you drown. But shame let me take that back because some other times we do go training, it’s only training related to disasters that we have never been to.’ (Participant 21, female, social worker)

Authors such as Huang et al. (2014), Matlakala et al. (2021) and Kranke et al. (2020) note that a lack of disaster management training among social workers hinders their ability to respond effectively to disasters. Kranke et al. (2020) further noted this to be emotionally burdening for social workers while additionally heightening their susceptibility to mental health issues, often even undermining their willingness to remain in the profession. However, Bauwens and Naturale (2017), in contrast to the previously cited studies, found that the majority of United States (US) social workers were well informed, adequately trained and experienced in the basic skills necessary to provide the social work services required during disasters. This suggests that adequate training and preparedness may positively influence social workers’ confidence, morale and willingness to remain engaged in disaster management. Although not specific to disaster social work, a study by Gad et al. (2022) identified the unavailability of professional training as one of the factors negatively affecting job satisfaction, while Boston (2009) noted professional development training to be useful in promoting retention among social workers and linked ongoing training to improved job satisfaction through the enhancement of existing professional skills. The reported deficiencies in training can be located in the exo-system, where limited training opportunities exist for practitioners. However, its consequences are evident in the micro-system through reduced confidence and uncertainty in providing services during disasters (Brakenhoff & Slesnick 2015; Zhang 2018).

### Role confusion because of an unclear scope of practice

Participants also experience role confusion because of the lack of a clear scope of practice for frontline social workers. As a result, they frequently found themselves in conflict with other stakeholders because of overlapping roles. This also left them feeling uncertain and unclear about their place within the broader context of disaster management. This is well illustrated by the quotes taken from interviews with two social workers below:

‘We cannot be a jack of all trades. That is our weakness in the Department of Social Development. We want to be involved in it all, and you cannot. The truth of the matter is, you cannot. The doctor knows that this is my part, and I stop here. From here, so and so comes in, and this is what they do, and we also need to understand our role, which most often is usually the last one.’ (Participant 17, female, social worker)

‘You find that in the municipality context, the roles of the social workers are not clear what is it that is expected of you in the context of responding to the disaster? That is very critical because it involves defining or having a definition of roles. It can make it easy for us to respond to disasters.’ (Participant 1, male, social worker)

Social work practitioners play a crucial role in providing care to people affected by disasters (Drolet et al. 2024; Hay & Pascoe 2021; Huang et al. 2014; Shokane 2019). However, working with multiple stakeholders in the absence of a clearly defined scope of practice makes navigating disaster management contexts difficult for practitioners (Sim et al. 2021). The overlapping roles and conflicting objectives of different stakeholders further create barriers for social workers during relief efforts (Cleary & Dominelli 2020). The absence of a clear scope of practice further subjects social workers to extremely high caseloads, dual roles, fatigue and minimal self-care as they are often expected to accommodate additional tasks that do not fall within their ambit of practice (Cederbaum 2022; Rai & Rammanohar 2023). Consequently, the limited understanding of social workers’ roles contributes to their underrecognition and limited opportunities to be fully engaged in disaster management (Drolet et al. 2024; Huang et al. 2014; Sim et al. 2021). In South Africa, this is further compounded by the vagueness of the legislature regarding the roles of human scientists, such as social workers, in disaster management, which also contributes to role conflicts (Nyahunda & Ncube 2025). The findings reflect both meso-system coordination challenges and macro-system misunderstandings of the role of social workers in disaster management, which contributed to uncertainty regarding practitioners’ scope of practice in the micro-system (Neal & Neal 2013; eds. Newman & Newman 2020). Despite this, practitioners continue to assume multiple roles and provide services, drawing on the profession’s broader values and principles located within the macro-system (Brakenhoff & Slesnick 2015; Zhang 2018).

### The lack of cooperation from community members and stakeholders

Lastly, the lack of cooperation from both community members and stakeholders (professionals and organisations) operating within the disaster management field was also identified as a significant challenge.

Due to a lack of understanding of social workers’ role, community members often had unrealistic expectations of them and were unwilling to engage when only psychosocial support, rather than material support such as housing, was offered. On the other hand, stakeholders were largely ineffective in executing their mandates, thereby burdening social workers on the ground and causing emotional and physical strain. Interviewees provided various examples drawn from their own experience:

‘One of my challenges will be that as different stakeholders arrive with various donations, when you get there with nothing materialistic to offer as a social worker, you are not taken seriously. The victims even say that you are nothing. At times when you call them to gather at the shelter, they first ask what you have to offer and tell you to leave them alone once they realise that you have nothing.’ (Participant 21, female, social worker)

‘Most of them are not patient. You know, as a social worker, you only have to provide psychosocial support, making sure that donated things are distributed accordingly. But people will be wanting houses, you know. And you cannot provide that. So, it becomes a challenge for them to stay in one space… So, people get frustrated, especially because they are used to their own spaces. So, when you want them to stay together, it becomes a very big challenge. And they become impatient because they want their own space. And when that doesn’t happen, they can even get violent.’ (Participant 19, female, social worker)

‘People are not interested in counselling when they are in the halls displaced. They just want to know what it is that you are offering to assist them. Do you have food for them? Do you have clothes for them? Do you have whatever for them? But if you’re just going to say, you’re coming to do a programme with them, like prevention programme, you’re coming to engage them, you’re coming to counsel them. They’re not interested, let’s be honest. They want people who are bringing something for them.’ (Participant 10, female, social worker)

Matlakala et al. (2021) note that disasters frequently leave victims and survivors without basic necessities, such as food and shelter. Beyond these, people lose homes, clothing, important documents, medication and possessions accumulated or inherited over a lifetime (Bragin 2011). In such instances, meeting immediate material needs takes priority over non-material services such as counselling. However, at such times, resource shortages make it difficult for social workers to fulfil their mandate and to provide material support (Huang et al. 2014; Matlakala et al. 2021; Rai & Rammanohar 2023). Consequently, communities may respond with scepticism, resistance, non-cooperation and even abuse (Cleary & Dominelli 2020).

Lack of cooperation from key stakeholders was also identified as a major challenge affecting social workers during disasters. The following extracts reveal how their absence frustrates the situations that social workers deal with in evacuation centres:

‘If sometimes people have been in the halls for a long time, you [*Department of Social Development*] DSD just go there with your yellow jacket to be visible, sitting at the tables and then people come, they ask you about houses. You don’t render the services. The people that are rendering those services are nowhere to be found. So sometimes, as social workers working under DSD, we find ourselves that we have to account for departments because most of them are not stationed there in disaster-stricken environments. Whereas with us, we expected to go there. But we can’t offer people what they need.’ (Participant 10, female, social worker)

‘It’s when there is no cooperation between disaster management, the Department of Social Development, or [*South African Social Security Agency*] SASSA, because these stakeholders were supposed to work together to assist the people in the shelter. So, if these stakeholders are pulling in different directions at the end of the day, the social worker needs to go and do the monitoring there, only to find that so and so was supposed to do a, b, c, d but failed to do so.’ (Participant 7, female, social worker)

Guerrero et al. (2023) identify stakeholder collaboration as key in shaping individual performance in multi-stakeholder disaster response systems. However, as Huang et al. (2014) have suggested, the lack of cooperation and coordination among government stakeholders and agencies in disaster-stricken areas is a key challenge for social workers. Similarly, Matlakala et al. (2021) and Cuadra (2015) report that poor interprofessional collaboration, often driven by stereotypical rivalries among disaster management stakeholders, undermines the effectiveness of frontline social workers. The lack of collaboration and cooperation among external stakeholders complicates the process of navigating the bureaucracy of different governmental and non-governmental organisations, making it more complex and time consuming for social workers (Madlah et al. 2023). The findings reflect meso-system collaboration challenges among relevant stakeholders and macro-system misunderstandings of the role of social work practitioners in disaster management. Although beyond the control of individual practitioners, these factors create barriers to practice and perpetuate existing challenges within the micro-system, thus shaping their experiences in disaster response (Crawford 2020; Neal & Neal 2013).

### Recommendations

Taking these challenges into consideration, the study recommends that a relevant disaster management policy specific to social workers be developed, mandating the provision of adequate resources and budget allocations to secure the equipment required for effective social work service delivery, including support staff and PPE. The proposed policy should also include mechanisms and protocols for monitoring the utilisation of these resources to ensure accountability and prevent misuse. Furthermore, specialised disaster management training, aligned with social workers’ roles and responsibilities (e.g. advanced crisis intervention and trauma counselling), should be provided continuously. Such training would ensure that social workers are equipped with up-to-date knowledge of disaster management, including emerging trends, and that their professional competencies to effectively provide services to those affected by disasters are refined. Lastly, a national scope of practice for social workers engaged in disaster management should be clearly defined and consistently communicated to both community members and relevant stakeholders. Such awareness should be regularly raised to help manage and further alleviate unreasonable expectations often placed on social workers during disasters and to eliminate role confusion among social workers and their counterparts within the disaster management field.

## Conclusion

Disaster frontline social workers operate within complex ecological environments where individual experiences are shaped by interactions arising across multiple systems. This aligns with ecological-systems theory’s assertion that human functioning is best understood through the dynamic interactions among multiple ecological systems rather than solely through individualistic explanations. Although practitioners demonstrate resilience and commitment to service delivery within the micro-system, these are often constrained by meso-, exo- and macro-systems challenges, some of which are compounded over time (chrono-system).

At the meso-system, the relationships and coordination among their primary organisations and other disaster management stakeholders influence service delivery effectiveness. At the exo-system level, organisational and broader structural factors, such as the lack of resources, inadequate training opportunities and the absence of a clear scope of practice, further shape frontline social work practice and their well-being. Additionally, the prevailing professional ideologies, value system and broader societal expectations at the macro-system influence how practitioners’ roles are constructed and understood. Lastly, chrono-system influences indicate that longstanding systemic challenges are exacerbated during disasters, often requiring practitioners to rely on personal resources to sustain service delivery, sometimes at the expense of their family relationships and personal well-being. Collectively, these interacting system factors contribute to psychological and emotional strain, physical exhaustion and reduced professional confidence and morale, thereby impacting both practitioner and service delivery. Overall, the findings underscore the importance of recognising the systemic challenges experienced by frontline social workers and prioritising their well-being within disaster management systems. This is significant as prolonged exposure to such challenges during disasters can contribute to long-term psychological distress and even exacerbate pre-existing vulnerabilities post-disaster.
